# The Carcinogenic Effects of Formaldehyde Occupational Exposure: A Systematic Review

**DOI:** 10.3390/cancers14010165

**Published:** 2021-12-29

**Authors:** Carmela Protano, Giuseppe Buomprisco, Vittoria Cammalleri, Roberta Noemi Pocino, Daniela Marotta, Stefano Simonazzi, Francesca Cardoni, Marta Petyx, Sergio Iavicoli, Matteo Vitali

**Affiliations:** 1 Department of Public Health and Infectious Diseases, Sapienza University of Rome, P.le Aldo Moro 5, 00185 Rome, Italy; carmela.protano@uniroma1.it (C.P.); vittoria.cammalleri@uniroma1.it (V.C.); robertanoemi.pocino@uniroma1.it (R.N.P.); daniela.marotta@uniroma1.it (D.M.); 2 Department of Human Anatomy, Histology, Forensic Medicine and Orthopedics, Sapienza University of Rome, P.le Aldo Moro 5, 00185 Rome, Italy; giuseppe.buomprisco@uniroma1.it (G.B.); stefano.simonazzi@uniroma1.it (S.S.); francesca.cardoni@uniroma1.it (F.C.); 3 Department of Occupational and Environmental Medicine, Epidemiology and Hygiene, INAIL, Via Fontana Candida 1, 00078 Monte Porzio Catone, Rome, Italy; m.petyx@inail.it (M.P.); s.iavicoli@inail.it (S.I.)

**Keywords:** formaldehyde, carcinogenicity, occupational exposure, cancer risk

## Abstract

**Simple Summary:**

Formaldehyde is a chemical compound present in many working activities and indoor workplaces. Occupational exposure occurs primarily by inhaling airborne formaldehyde, but it can also be absorbed through the skin or ingested. The International Agency for Research on Cancer (IARC) classified formaldehyde as a Group 1 carcinogen for humans in 2004, based on toxicological data and epidemiological evidence obtained in workplaces, all published before that year. Over the last two decades, many new studies in this field have been published, providing updated findings. The aim of the present systematic review was to synthetize the results of epidemiological studies in occupational settings carried out in the last 20 years and to evaluate whether the IARC classification was confirmed by further studies. Our results show that the evidence of correlation between formaldehyde occupational exposure and the occurrence of cancer is limited.

**Abstract:**

Background: Formaldehyde, classified as a carcinogen in 2004, as of today is widely used in many work activities. From its classification, further studies were performed to evaluate its carcinogenicity. The aim of the systematic review is to update the evidence on occupational exposure to formaldehyde and cancer onset. Methods: The review, in accordance with the PRISMA statement, includes articles in English reporting original results of studies conducted on workers exposed to formaldehyde, considering all types of cancer, published from 1 January 2000 to 30 July 2021 and selected from the Pubmed and Scopus databases. The studies’ quality was assessed by the Newcastle–Ottawa Scale. Results: A total of 21 articles were included, conducted in different European, American, and Asian countries. The most investigated occupational areas are those characterized by a deliberate use of formaldehyde. Some studies evaluated all types of cancer, whereas others focused on specific sites such as thyroid and respiratory, lymphohematopoietic, or central nervous systems. The results showed weak associations with lung cancer, nasopharyngeal cancer, leukemia, and non-Hodgkin’s lymphoma. Conclusions: The results demonstrate the need for further original studies carried out on representative samples of workers exposed to measured levels of FA. These studies should be designed to reduce the bias due to co-exposure to other carcinogens.

## 1. Introduction

Formaldehyde (FA) is a chemical compound naturally occurring in the atmosphere, in some foods, and in the organisms of mammals as a product of oxidative metabolism and, thus, is considered a ubiquitous pollutant. In addition to these sources, FA can be released in the environment through combustion processes or by degradation of some hydrocarbons such as methane. Besides, due to its chemical–physical characteristics, FA is widely applied in many productive processes, such as the construction materials industry, the chemical industry (resins, paintings, etc.), the wood-processing and furniture industry, the food industry, biomedical laboratories, gross anatomy rooms, handicrafts, etc. [1]. Consequently, many types of occupational activities determine FA exposure. Driscoll et al. [2] conducted a study based on data obtained from the Australian Workplace Exposures Study about the prevalence and patterns of exposure to 38 known or suspected carcinogens, including FA, among the Australian working population. As a result, 2.5% of the workers were likely to have been exposed to FA. The main working activities that exposed them to this chemical were the processing of chipboards or plywood panels for carpentry, building maintenance, and sanding before painting. The other workers most exposed were firefighters [3,4,5], healthcare workers [6], and beauticians. FA has also been detected in restaurants [7,8] when grilling dishes and adding sauces, in copy shops [9,10], in gardening [11], in the agri-food sector [12,13], in veterinary clinics, in embalming laboratories, in industrial launderings, etc. Besides, FA is frequently found in building environments, posing at potential risk of exposure to all indoor workers [14,15,16,17,18,19].

Exposure occurs primarily by inhaling airborne FA, but it can also be absorbed through the skin or ingested. The International Agency for Research on Cancer (IARC) in 2004 concluded that there was sufficient evidence of the carcinogenicity of FA for humans to reclassify FA from Group 2A (probably carcinogenic to humans) to Group 1 (carcinogenic to humans) [20]. In the subsequent monograph n. 100 of 2012, in summary, the IARC confirmed that there was sufficient epidemiological evidence that FA causes tumors of the nasopharynx, insufficient evidence of a causal relationship with leukemia, and limited epidemiological evidence for nasal sinus cancer [21]. EU Regulation 2015/491 also imposed the reclassification of FA from suspected carcinogen to carcinogen for humans in category 1B (i.e., it can cause cancer) on the basis of sufficient evidence both in humans [22,23] and in experimental animals [24,25]. However, all the scientific evidence that led to these classifications date back to before 2005. Besides, most of the studies on the relationship between FA and cancer were in vitro experiments demonstrated the effects on culture cells. Researchers have found many cellular damages, like DNA and RNA alterations [26,27], the onset of DNA–protein crosslinks, changes in p53 protein expression [28], and histone modifications [29]. On the other hand, epidemiological studies have not been able to confirm this association. In addition, several previous reviews investigated the relationship between occupational FA exposure and the onset of specific cancers, often obtaining conflicting conclusions [30,31,32,33,34]. However, no recent systematic review has looked into the relationship between occupational exposure to FA and the occurrence of cancer, except one published 15 years ago that concluded that there was no appreciable excess risk for cancers of the oral cavity and pharynx, sinus and nasal cavity, nasopharynges, and lung [35].

The aim of this systematic review is to update the scientific evidence on the relationship between occupational exposure to FA and the occurrence of all kinds of cancer evaluated by epidemiological studies performed on humans. The results of the review might help to confirm the evidence already produced by previous studies, or to highlight the need to review the current classification and/or to carry out new studies.

## 2. Materials and Methods

The presentation of this systematic review is in accordance with the latest version of the PRISMA statement [36]. We started the review process before the publication of the PRISMA Statement 2020; for this reason, the first steps of the review were conducted following the old version (PRISMA Statement 2009), which was less stringent in the “protocol and registration” item, reporting the following sentence “Indicate if a review protocol exists, if and where it can be accessed (e.g., Web address), and, if available, provide registration information including registration number)”. Thus, initially we did not register the protocol in any database and, then, it was too late to do it because the protocol registration must be performed before the start of the review process. Zotero citation management software (RRID:SCR_013784) was used to identify any duplicates and to manage and screen the identified records.

### 2.1. Literature Research

The review includes articles published in the last 20 years, from 1 January 2000 to 31 July 2021, on the databases Pubmed and Scopus. The search strategy used a combination of controlled vocabulary and free text terms based on the following keywords: “formaldehyde”, “cancer”, “tumor”, “neoplasm”, “occupational”, and “exposure”. Additionally, a hand search of the reference lists of the selected articles was carried out for a wider analysis. Four independent reviewers (V.C., R.N.P., D.M. and G.B.) performed the search, reading the titles and abstracts of the articles identified by the search strategy.

During the multi-step exclusion process, any disagreement on the studies was discussed until consensus. The process was supervised by other investigators (C.P. and M.V.).

Figure S1 (Supplementary Materials) shows the flow chart summarizing the selection steps for the systematic review.

### 2.2. Inclusion and Exclusion Criteria

The review included only studies in which the participants were classified as “exposed to formaldehyde.” The exposure assessment was considered acceptable if performed by direct (personal or environmental) sampling of FA, occupational history data, or job exposure matrix. Cancers were classified using the International Classification of Diseases, Tenth Revision (ICD-10).

Only studies involving humans (men and/or women) exposed to FA in occupational settings, reporting results for any kind of cancer and published in peer-reviewed journals, were selected. Searches providing no information about the exposure assessment method or with a self-assessment by the participants were excluded. Besides, we excluded reviews, editorial articles, individual contributions (i.e., conference speeches), and purely descriptive studies published in scientific conferences without any quantitative or qualitative conclusions. Finally, articles published in languages other than English were excluded.

### 2.3. Data Analysis

From each study included in the review, the following data were extracted: publication year, exposure time period, study design, working population studied, cancer type (ICD-10 classification), exposure assessment, and main conclusions.

### 2.4. Quality Evaluation

Four different reviewers (V.C., R.N.P., D.M. and G.B.) assessed the methodological quality of the selected studies with a specific rating tool, the Newcastle–Ottawa Scale (NOS), adapted for evaluating case-control, cross-sectional, and cohort studies [37]. It is divided into eight categories checking three quality aspects: selection, comparability and outcome/exposure; scores range from 0 to 9. The quality of a study was considered to be high if the NOS score was 7 to 9, intermediate if the NOS score was 4 to 6, and low if it was 0 to 3.

## 3. Results

In total, we recovered 1029 studies from all searched databases (*n* = 629 from Scopus and *n* = 400 from Pubmed) and, after applying filters by automation tools, 390 articles remained. Out of the remaining 390 papers, 350 were excluded after removing duplicates. Successively, one more paper was removed after reading the abstract. Then, the full texts of 39 studies were checked and evaluated considering the inclusion/exclusion criteria. A total of 14 papers were then excluded because they did not fit the inclusion criteria. Besides, eight articles were based on the same studied cohort; thus, we considered only the most recent, excluding those previously published. Six articles were found via citation search and four were included after checking their eligibility, whereas two were discarded due to the difficulty of extrapolating FA exposure. At the end of the process, 21 articles were included in the systematic review [38,39,40,41,42,43,44,45,46,47,48,49,50,51,52,53,54,55,56,57,58]. The PRISMA Flow Diagram is available as Supplementary Materials (Figure S1).

Table 1 shows the characteristics of the studies included, with reference to country, workers’ gender, sample size, working context, study period, smoking adjustment, exposure assessment, cancer type, and main conclusions.

### 3.1. Characteristics of the Included Studies

The studies included were conducted on almost all continents, with six from Europe, nine from North America, four from Asia, one from South America, and one multicenter study. Most (11 studies) involved both sexes, seven involved only males, and three only females. In total, 11 case-control studies, eight cohort studies, and two case-cohort studies were considered. Industry and manufacturing were the most examined working contexts, in particular the sectors of chemical, plywood, and textile production. Therefore, the majority of the studies included (n. 11) regarded workers exposed to FA in several working contexts and workplaces. Twelve studies considered smoking a confounding factor and adjusted the results accordingly. Only three research groups performed the direct exposure assessment through personal or environmental sampling; the others assessed the exposure level indirectly by job exposure matrix or occupational history data. Five studies looked for the relationship between FA exposure and the onset of any cancer, seven evaluated the onset of upper airway cancers, four focused on lung cancer, two focused on lympho-hematopoietic cancers, one focused on thyroid cancer, and one focused on meningioma. The sample size was very variable, ranging from two cases and five controls in the smallest case-cohort study to a cohort of 1.2 million workers in the study by Siew et al. [51].

### 3.2. Scoring Results

The median NOS score of the included studies was 7, thus indicating a high average quality level. Table 2 shows the results of the scoring method applied to each study included in the review, with reference to publication year, study design, and main statistical results achieved (expressed as odds ratio, hazard ratio, relative risk, or standardized mortality ratio and with a 95% confidence interval).

## 4. Discussion

We performed a systematic review on the association between FA occupational exposure and the occurrence of cancer in potentially exposed workers.

Previous studies about FA and cancer risk suggested a modest excess of risk for nasopharyngeal cancer [59], but the studied cohort of workers was co-exposed to several other chemicals, resulting in additive and/or synergic effects or misleading results. Despite this, the findings were included by the IARC in its evaluation, even if subsequent analysis revealed no statistical significance of these results and highlighted the inappropriateness of the adopted exposure assessment approach [60].

Among the studies included in our review, we found a direct assessment of the exposure levels of FA only in three papers. In the other cases, the exposure assessment was indirectly extrapolated considering the length of exposure and the type of activities performed (e.g., job exposure matrix). Most of the studies included in this review dealt with occupational settings, characterized by a deliberate use of FA as a component of the production cycle. Those were mainly represented by chemical industries dedicated to the production of plastics, fiberglass, paints, etc.; it is reasonable to imagine that in such contexts the levels of exposure to FA were particularly high. Three studies were carried out in textile-/garment-producing plants, where FA is used to give resistance to the folds of clothing fabrics and for the processing of leathers. Another sector where this substance is widely used is that of woodworking and furniture making. In fact, FA, together with resins, gives strength and resistance to chipboard panels. FA is also widely used in the medical field: in the operating room it was used to disinfect instruments because of its high antibacterial power, and even today, it is used to avoid the deterioration of human tissues that must undergo histopathological analyses. Despite that, very few studies concerned the health sector, or the agri-food industry, where FA is used as a preservative. That is quite surprising, considering that there is much research about the occupational exposure to FA in pathological anatomy settings and sector rooms [61,62,63] that stress the needing for adequate preventive measures for workers [64].

Although the genotoxicity and immunotoxicity of FA is well known and has been demonstrated by several studies regarding its influence on DNA and pro-oxidative effects on cells [28,65,66,67,68,69,70,71,72], the evidence from human studies and diagnosed cancers is much less consistent [73]. Most of the studies included in this review focus on upper-airway neoplasms (ICD-10 codes: C10–C14 and C30–C33), as mentioned previously. In fact, the main way of entry of this substance into the body is by inhalation. Five studies explored the relationship between FA occupational exposure and the onset of lung cancer (ICD-10 code: C34). Their findings contrasted with each other: some did not provide evidence of a carcinogenic effect on the lungs [42,51,52], whereas others found a correlation [44,49]. These last studies, however, were performed on a very small sample and present several limitations (e.g., self-reported data on exposure levels). A recent meta-analysis by Kwak et al. [31] concluded no significant increase in the risk of lung cancer, even considering only groups of highly exposed workers. The small study sample of the study by Checkoway et al. about lung cancer was also checked for thyroid cancer, with some relationships found but with the same, considerable, limitations [46]. In 2012, the IARC affirmed that there was strong but insufficient evidence of a causal relationship with leukemia. Two studies included in our review regarded the relationship between FA exposure and lympho-hematopoietic cancers (ICD-10 codes: C81–C96), but no association was observed for all leukemias [56], except for a small and weak association with non-Hodgkin lymphoma [48] and myeloid leukemia [50]. This is consistent with the results of other previous studies [30,33]. Five of the included publications evaluated the effects of FA occupational exposure on the onset of any kind of cancer. These were large cohort studies, carried out in Europe and the USA in industrial contexts, and almost all concluded no positive association with FA exposure and the mortality from any cancer, and very limited evidence with NPC and leukemia [43,53,54,55,56]. The most recent research included, published in 2018, was a multicenter study about FA and meningioma. Meningiomas are tumors that develop from the meninges, tissues that surround the outside of the brain and account for about 30% of brain tumors. Although benign, they are dangerous because dysphagia, dysarthria, ocular motility disorders, and facial numbness can occur. Intracranial hypertension, focal seizures, lack of strength, and balance and gait disturbances may also sometimes occur. The study concluded that FA did not provoke excess risks of meningioma [58].

The present systematic review has some limitations. First of all, we considered only papers published in the last 20 years, but this choice was driven by the aim of the present systematic review. Secondly, we considered only articles published in the English language, excluding a priori potentially useful results published in other languages. Finally, we did not perform a formal meta-analysis because the studies included in the review were different in terms of exposure assessment methodologies, kind of cancers considered, and study design. For this reason, statistical heterogeneity and publication bias were not evaluated. Our choice is well supported by a very recent official statement by Cochrane on the opportunity for performing a meta-analysis when data are heterogeneous: “Meta-analysis should only be considered when a group of studies is sufficiently homogeneous in terms of participants, interventions and outcomes to provide a meaningful summary. It is often appropriate to take a broader perspective in a meta-analysis than in a single clinical trial. A common analogy is that systematic reviews bring together apples and oranges, and that combining these can yield a meaningless result” [74].

## 5. Conclusions

FA has been classified by the IARC as a Group I carcinogen since 2004; this classification was based on evidence obtained in preceding years. Reviewing the scientific literature published in the last 20 years, we found at least 21 additional epidemiological studies on the association between occupational exposure to FA and cancer onset. This finding indicates the need for an update of the FA classification based on the new evidence. On the other hand, the results of the examined papers do not completely confirm the IARC classification of FA and give contrasting results. Thus, it is essential to perform further original studies carried out on representative samples of workers exposed to measured levels of FA. These studies should be designed to reduce bias as much as possible due to co-exposure to other carcinogens.

## Figures and Tables

**Table 1:** Characteristics of the studies (*n* = 21) included in the systematic review.

Reference [n.]—Country	Workers’ Gender	Sample Size *	Working Context	Study Period	Risk—Adjusted for Smoking Habits	Exposure Assessment	Cancer Type (ICD-10)	Main Conclusions
[38]—Malaysia	Both	49 cases, 49 controls	Chemical, plywood, and textile industries	1990–1992	Yes	Air sampling **	Nasopharyngeal cancer (C11.9)	No association was found between nasopharyngeal carcinoma and FA.
[39]—USA	Both	79 cases, 79 controls	Various	1987–1993	Yes	Occupational history data	Nasopharyngeal cancer (C11.9)	Results from this study support the hypothesis that occupational exposure to FA increases risk of NPC. The association between risk of NPC and potential exposure to FA was stronger among cigarette smokers.
[40]—France	Men	Laryngeal: 102 cases, 85 controls Hypopharyngeal: 83 cases, 85 controls	Various	1987–1991	Yes	Job exposure matrix	Laryngeal and hypopharyngeal cancer (C10.9)	Exposure to FA was associated with an increased risk of hypopharyngeal cancer. No association with laryngeal cancer was found.
[41]—Taiwan	Both	74 cases, 41 controls	Various	1991–1994	Yes	Occupational history data	Nasopharyngeal cancer (C11.9)	There was some evidence of increasing risk of NPC with increasing years of exposure to FA, but the observed trend did not achieve statistical significance.
[42]—Northern Europe	Men	108 cases, 398 controls	Vitreous fiber-producing plants	1971–1996	Yes	Occupational history data	Lung cancer (C34)	This study provides no evidence of a carcinogenic effect on the lungs from FA exposure.
[43]—USA	Both	11,039	Various	1955–1998	Not	Personal sampling among workers (1981 and 1984) **	All cancers (C00–C97)	Results support a possible relation between FA exposure and myeloid leukemia mortality. Non-significant excesses in mortality were observed among FA-exposed workers for several other cancers.
[44]—Uruguay	Men	32 cases, 65 controls	Agricultural workers, histology technicians, medical personnel, and foundry workers	1994–2000	Yes	Occupational history data	Lung cancer (C34)	Constant exposure to FA was significantly associated with an increased OR of adenocarcinoma of the lung.
[45]—Central and Eastern Europe	Men	18 cases, 30 controls	Various	1999–2002	Yes	Occupational history data	Laryngeal cancer (C32) Hypopharyngeal cancer (C12, C13)	No overall association was found between FA and laryngeal cancer.
[46]—China	Women	2 cases, 11 subcohort non-cases	Textile industries	1989–1998	Not	Historical measurements data	Thyroid cancer (C73)	Associations were observed between thyroid cancer and employment in jobs with 10 or more years of FA exposure.
[47]—USA	Both	7345	Plastic-producing plants	1979–2003	Yes	Occupational history data	Nasopharyngeal cancer (C11.9)	Overall, the pattern of findings suggests that the large, persistent nasopharyngeal and other PC excesses observed were not associated with FA exposure. Interaction models suggest that NPC and AOPC risks were not elevated in subjects exposed only to FA.
[48]—USA	Women	201 cases, 203 controls	Various	1996–2000	Yes	Job exposure matrix	Non-Hodgkin lymphoma (C85.90)	Exposure to FA was found to be associated with an increased risk of NHL in our study, but the risk was mainly for those with a low exposure intensity or probability.
[49]—China	Women	2 cases, 11 subcohort non-cases	Textile industries	1989–1998	Yes	Job exposure matrix	Lung cancer (C34)	Exposures to silica and FA may have increased lung cancer risk. This observation was based on very small numbers of exposed workers.
[50]—USA	Men	144 cases, 210 controls	Funeral industry workers	1960–1986	Yes	Historical measurements data	Nasopharyngeal cancer (C11.9) Lympho-hematopoietic cancers (C81–C96) Myeloid leukemia (C92.90) Brain cancer (C71)	The duration of embalming practice and related FA exposure in the funeral industry were associated with statistically significantly increased risk for mortality from myeloid leukemia.
[51]—Finland	Men	1, 2 mln	Various	1971–1995	Yes	Job exposure matrix	Nasopharyngeal cancer (C11.9) Lung cancer (C34)	The results are inconclusive, but FA did not appear to increase risk in any way whatsoever for nasal, nasopharyngeal, or lung cancer.
[52]—Canada	Both	347 cases, 325 controls	Various	1979–2002	Yes	Job exposure matrix	Lung cancer (C34)	No marked increases in lung cancer risk related to workplace FA exposure were observed.
[53]—USA	Both	11,043	Garment-manufacturing facilities	1985–2008	Not	Personal sampling **	All cancers (C00–C97)	We continue to see limited evidence of an association between FA and leukaemia. We did not find solid evidence of increased mortality from other lympho-hematopoietic cancers and a priori solid cancers with FA exposure.
[54]—USA	Both	25,619	Various	1950–2004	Not	Historical measurements data	All cancers (C00–C97)	For all cancer, solid tumors, and lung cancer, the mortality among exposed workers was high, but internal analyses revealed no positive associations with FA exposure. Consistent with previous analyses of this cohort, this update continues to suggest a link between FA exposure and nasopharyngeal cancer.
[55]—UK	Men	14,008	Chemical industries	1941–2012	Not	Occupational history data	All cancers (C00–C97)	Our results provide no support for an increased hazard of myeloid leukemia, nasopharyngeal carcinoma, or other upper airway tumors from FA exposure. These results indicate that any excess risk of these cancers, even from relatively high exposures, is at most small.
[56]—Italy	Both	2750	Laminated plastic factories	1947–2011	Not	Occupational history data	All cancers (C00–C97)	We found no meaningful excess mortality from any lymphohematopoietic nor other neoplasms, except possibly for nasopharyngeal cancer.
[57]—USA	Both	25,619	Various	1930–2004	Not	Historical measurements data	Lympho-hematopoietic cancers (C81–C96)	No association between cumulative FA exposure and mortality from all leukemias combined was observed for the entire cohort.
[58]—Multicenter study	Both	116 cases, 278 controls	Various	1945–2003	Not	Job exposure matrix	Meningioma (D32.9)	This study shows an increased risk in relation to FA based mainly in women in relation to a duration of exposure of more than 15 years and highest cumulative exposure, although neither of the trends was statistically significant.

* Both cases and controls exposed to formaldehyde; ** direct exposure assessment.

**Table 2:** Scoring results of the included studies in relation to study design, year of publication, and statistical results achieved.

Reference [n.]—Year	Study Design	Statistical Results	NOS Score
[38]—2000	Case-control	Nasopharyngeal cancer: OR: 0.88 (CI: 0.70–1.12)	7
[39]—2000	Case-control	Nasopharyngeal cancer: OR: 1.3 (CI: 0.80–2.1)	7
[40]—2000	Case-control	Hypopharyngeal cancer: OR: 1.35 (CI: 0.86–2.14) Oropharyngeal cancer: OR: 1.14 (CI: 0.76–1.70)	6
[41]—2001	Case-control	Nasopharyngeal cancer: OR: 1.4 (CI: 0.93–2.2) *	6
[42]—2002	Case-control	Lung cancer: OR: 1.33 (CI: 0.76–2.34)	7
[43]—2004	Cohort	All cancers: SMR: 0.89 (CI: 0.82–0.97) Myeloid leukemia: SMR: 1.44 (CI: 0.80–2.37)	7
[44]—2005	Case-control	Lung cancer: OR: 1.7 (CI: 1.1–2.8)	6
[45]—2006	Case-control	Laryngeal cancer: OR 1.68 (CI: 0.85–3.31) *	6
[46]—2006	Case-cohort	Thyroid cancer: HR: 8.33 (CI: 1.16–60)	7
[47]—2007	Cohort	Nasopharyngeal cancer: SMR: 4.43 (CI: 1.78–9.13) Other pharynx cancers: SMR: 1.71 (CI: 1.01–2.72)	7
[48]—2008	Case-control	Non-Hodgkin lymphoma: OR: 1.3 (CI: 1.0–1.7)	7
[49]—2009	Case-cohort	Nasopharyngeal cancer: OR: 0.1 (CI: 0.01–1.2) Lympho-hematopoietic cancers: OR: 0.9 (CI: 0.4–2.1) * Myeloid leukemia: OR: 3.9 (CI: 1.2–12.5) Brain cancer: OR: 1.9 (CI: 0.7–5.3) *	
[50]—2011	Case-cohort	Lung cancer: HR: 2.10 (0.40–11.00)	8
[51]—2012	Cohort	Nasopharyngeal cancer: RR: 0.87 (CI: 0.34–2.20) Lung cancer: RR: 1.18 (CI: 1.12–1.25)	8
[52]—2013	Case-control	Lung cancer: OR: 1.06 (CI: 0.89–1.27)	7
[53]—2013	Cohort	All cancers: SMR: 0.96 (CI: 0.90–1.02)	7
[54]—2013	Cohort	All cancers: SMR: 1.08 (CI: 1.05–1.12)	7
[55]—2014	Cohort	All cancers: SMR: 1.10 (CI: 1.06–1.15)	7
[56]—2014	Cohort	All cancers: SMR: 79.8 (CI: 67.5–93.6)	6
[57]—2016	Cohort	Lymphohematopoietic cancers: SMR: 2.07 (CI: 1.22–3.49)	8
[58]—2018	Case-control	Meningioma: OR: 1.02 (CI: 0.80–1.29)	7

*: Not statistically significant; CI: 95% confidence interval; OR: odds ratio; HR: hazard ratio; RR: relative risk; SMR: standardized mortality ratio.

## Data Availability

Data are provided as tables and figures directly within the manuscript, and raw data are available via e-mail upon request to the corresponding author.

## Supplementary Materials

The following are available online at https://www.mdpi.com/article/10.3390/cancers14010165/s1, Figure S1: PRISMA flow diagram.
