# Supplementary material for: The Carcinogenic Effects of Formaldehyde Occupational Exposure: A Systematic Review
Source: Cancers (Basel). 2021 Dec 29;14(1):165. doi: 10.3390/cancers14010165 (PMC8749969; doi:10.3390/cancers14010165)
Supplement: Supplementary file 1 [file cancers-14-00165-s001.zip › cancers-1484336-supplementary.pdf]

PRISMA 2020 flow diagram for new systematic reviews which included searches of databases, registers and other sources

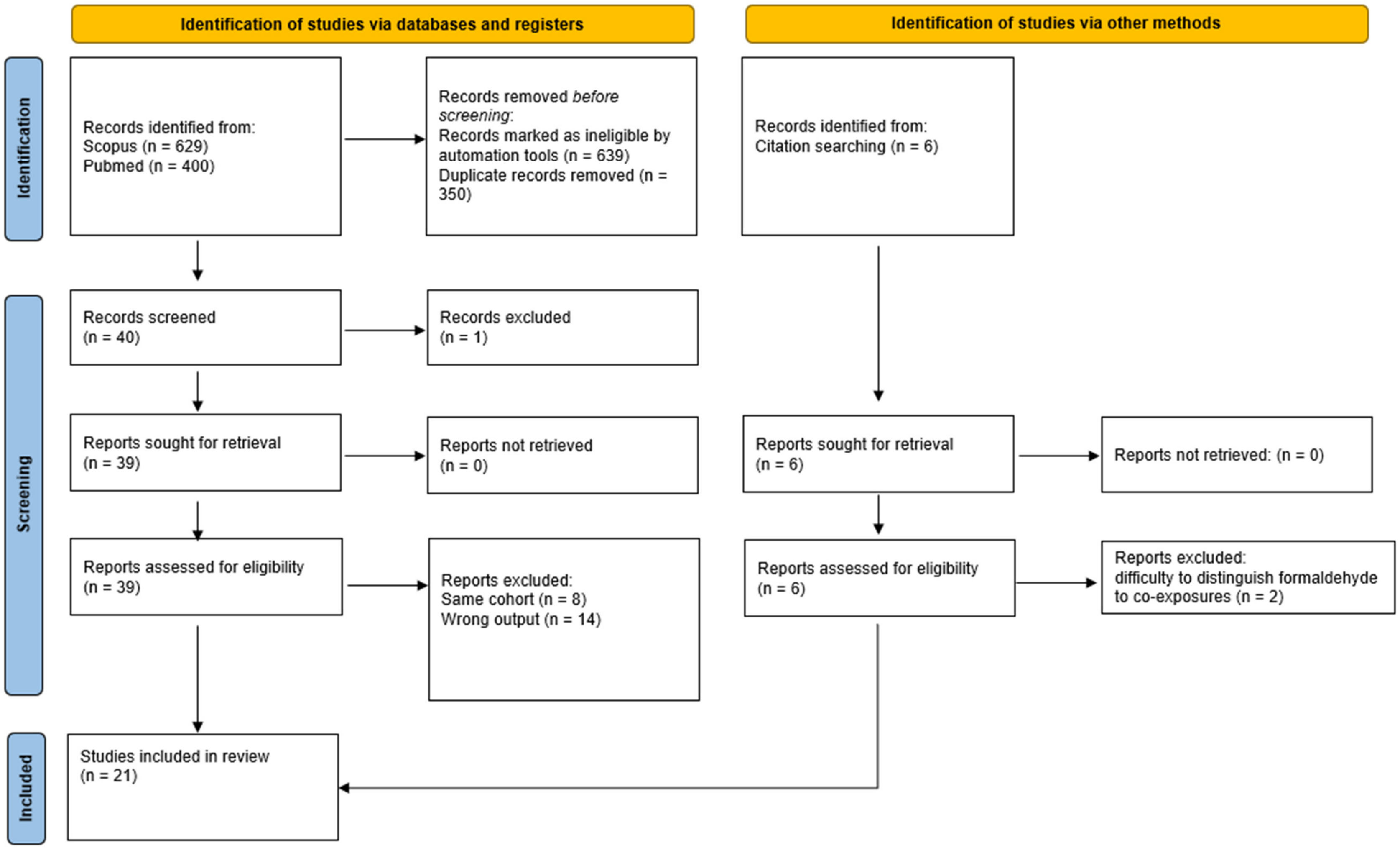

Figure S1. PRISMA flow diagram.

From: Page, M.J.; McKenzie, J.E.; Bossuyt, P.M.; Boutron, I.; Hoffmann, T.C.; Mulrow, C.D.; Shamseer, L.; Tetzlaff, J.M.; Akl, E.A.; Brennan, S.E.; et al. The PRISMA 2020 statement: an updated guideline for reporting systematic reviews. BMJ 2021, 372, n71, doi: 10.1186/s13643-021-01626-4.
